# Cognitive Analytic Therapy for Functional/Dissociative Seizures in an Adolescent: Case Report and Mixed-Methods Single-Case Evaluation

**DOI:** 10.3390/reports8020093

**Published:** 2025-06-11

**Authors:** Andrew Horan, Stephen Kellett, Chris Gaskell, Conor Morris

**Affiliations:** 1 Rotherham Doncaster and South Humber NHS Foundation Trust, South Yorkshire S26 4TH, UK; andrew.horan2@nhs.net (A.H.); christopher.gaskell@combined.nhs.uk (C.G.); 2 North Staffordshire Combined NHS Foundation Trust, Staffordshire, Stoke-on-Trent ST4 8HH, UK; 3 Clinical and Applied Psychology Unit, University of Sheffield, Sheffield S10 2TN, UK; 4 Private Practice, London E10 6NJ, UK

**Keywords:** functional neurological disorder, dissociative/functional seizures, brief cognitive analytic therapy, case report

## Abstract

**Background and clinical significance**: Functional/dissociative seizures (FDSs) in adolescents are paroxysmal events which superficially resemble epileptic seizures or syncope. This study evaluated the effectiveness of brief cognitive analytic therapy (CAT). **Case presentation**: The patient was a 17-year-old white cisgender male with a diagnosis of non-epileptic attack disorder. The functional/dissociative seizures were treated with 8-session CAT, with follow-up at 5 weeks. Two target problems (TPs) and associated target problem procedures (TPPs) were rated for recognition and revision at each session and at follow-up. An A-B-C-FU single-case experimental evaluation of the TP/TPPs was conducted. Nomothetic outcome measures (DES-2 and RCADS) were administered at session 1, session 8, and at follow-up, and the YP-CORE and the Session Rating Scale were completed at each session. The patient was independently interviewed using the Change Interview 13 weeks after completing therapy. The results show that CAT effectively increased the recognition and revision of TPs/TPPs, four specific changes occurred (including cessation of functional seizures). There were pre–post reliable and clinically significant improvements to psychological wellbeing, but these were not maintained at follow-up. **Conclusions**: This study indicates that CAT was a partially effective intervention. The use of CAT as a treatment for FND in adolescents holds promise, but more research is needed.

## 1. Introduction and Clinical Significance

Functional neurological disorder (FND) is as a ‘multi-network brain disorder’ [1] which incorporates a heterogeneous range of neurological symptoms or phenotypes (e.g., movement disorder, seizures, cognitive and sensory problems). Unlike traditional neurological conditions, FND does not include underlying changes to brain structure(s). In children and adolescents, functional movement disorder (FMD) and functional dissociative/seizures (FDSs) are the most common forms of FND [2]. FMD symptoms can include functional limb weakness/paresis, functional movement disorders, swallowing difficulties, regurgitation, and cough. FDSs are paroxysmal events that superficially resemble epileptic seizures or syncope with common symptoms, including episodic unresponsiveness, limb shaking, loss of muscle tone, faint-like events, and altered awareness, and so are highly heterogeneous in presentation [3]. To date, there has been more research conducted on adult FND, with childhood and adolescent FND having a less developed evidence base [4]. Misdiagnosis, inappropriate or unnecessary intervention, and risk of iatrogenic harm are therefore unfortunately also features of this evidence base [5].

The prevalence of FND in children and adolescents has been estimated at 1.3–6.0 per 100,000 [2,6,7]. FND is therefore a common reason for children and adolescents to see a neurologist, representing 10% of referrals to paediatric neurology clinics [8] and 20% to specialist epilepsy clinics [9]. Although FND can vary in age of onset, the average modal age of onset is 15 years [10] and is more common in female adolescents [6]. Whilst functional disorders are therefore fairly common presentations in neurology clinics, this has only recently been reflected in classification systems [11]. A range of dissociative neurological symptom disorders were introduced for the first time in the 11th revision of the World Health Organization’s International Classification of Diseases (ICD-11) [12]. The impact of FND on children and adolescents is significant and can include struggling to cope with multiple somatic symptoms, and with the necessary high service utilisation then interfering with personal development [4]. Asadi-Pooya et al. [13] evidenced that FND also exerts systematic pressure on the family unit beyond the marked emotional suffering of the child or adolescent.

Whilst FND can only be reliably diagnosed by neurologists, the burden of intervention tends to fall on psychological therapists. Interventions for child and adolescent FND have fallen into three camps: (a) outpatient 8-session retraining and control therapy (ReACT), (b) other outpatient cognitive–behavioural therapies (generally 10–16 sessions), and (c) 4-week multidisciplinary inpatient rehabilitation [14]. In a narrative review of these interventions [14], there was seizure cessation at the end of treatment in 63–95% of cases. Caution needs to be applied to this estimate as it was not based on a proportional meta-analytic method. Spontaneous remission, for example, is also possible and tends to occur post-diagnosis [15]. Whilst psychological interventions for FND seem to improve prognosis, the evidence base is clearly in need of further development [16], and this includes evaluations of other psychotherapeutic models beyond ReACT, CBT, and inpatient rehabilitation. Due to the time it takes to deliver a psychological intervention, and the risk of creating long wait-times for psychological interventions [17], the efficacy and acceptability of brief interventions are of particular interest in FND. This current study is therefore the first to evaluate the effectiveness of brief (i.e., 8-session) cognitive analytic therapy [CAT] [18] in a confirmed case of FDS in an adolescent.

The evidence base for CAT in the treatment of FND comprises two uncontrolled case reports of outpatient treatment with adults [19,20] and one mixed-methods single-case experimental design with an inpatient adult [21]. The uncontrolled case studies showed clinical improvement. The SCED showed partially effective therapy with improved self-awareness and psychological well-being across phases of therapy; however, there was no reduction in FDS frequency. SCED enables increased methodological rigour in the evaluation of clinical effectiveness by utilising experimental methodologies and associated phase comparisons of intensively measured idiographic and nomothetic outcome measures [22]. This current study hypothesised that there would be (a) a significant increase in recognition and revision of TPs/TPPs according to intervention phase; (b) a reliable and clinically significant pre-post improvement in depression, anxiety, distress, and dissociation on the nomothetic outcome measures that would be maintained at follow-up; and (c) the Session Rating Scale would significantly increase following case reformulation.

## 2. Case Presentation

The patient was a 17-year-old white cisgender male who had received a diagnosis of non-epileptic attack disorder (NEAD) and Tourette syndrome from a consultant neurologist. This diagnosis would fit under the broad category of FND [23]. NEAD is a term that has historically been used (among others) in reference to FDS [24]. The preference for FDS here is in accordance with that favoured by the International League Against Epilepsy. The patient’s past medical history included bladder overactivity and ongoing investigations for Marfan syndrome and arthritis. The neurological diagnosis of the patient noted the following aspects and symptoms: onset episodes at age 11 years and characterised by collapses and shaking movements. Attacks tended to involve collapsing, shaking violently, or becoming stiff and floppy, with a sense of anxiety and dread prior to the episodes, and the prodromal symptoms lasted between 5 and 120 min. The duration of the collapses varied between 10 and 360 min, depending on the duration of the prodromal symptoms. Following events, the patient reported fatigue, slurred speech, and difficulty walking. No incontinence or tongue-biting occurred during such episodes. The episodes were triggered and exasperated by (a) stress, with higher frequency in more stressful periods (e.g., exams) and (b) relational conflict (e.g., difficulties with the parents, a relationship breakup, and bullying at college). In more recent years, the patient experienced episodes in which he remained fully conscious, with duration ranging from brief events (lasting 5–20 min) to more prolonged episodes (lasting up to 120 min). Physical examinations showed no evidence of motor weakness or sensory deficits. Gait, muscle tone and cranial nerve function were all within normal limits and visual fields were normal. EEG, MRI, or video-telemetry tests were not part of the neurological assessment.

The patient was born in the UK and lived in the north of England initially in a nuclear family with an older sister. There were no concerns regarding early development. There was domestic violence between the parents during his early years, until the parents separated when the patient was 5 years old. He then resided with his mother but remained in contact with his father and visited him frequently. The mother experienced significant childhood trauma and adversity and had chronic mental health problems herself and was receiving ongoing treatment for anxiety and depression. The patient’s memories of his early years were that family life was highly unpredictable, and he recalled several events in which his mother was extremely emotionally dysregulated. He described himself as an angry child. In terms of education, he was regarded as academically average and was bullied at both primary and secondary school. The family received support from ‘Early Help’, a children’s services preventative intervention delivered to families where concerns do not meet the threshold for a statutory intervention (i.e., child protection). At assessment, the client lived with his mother, stepfather and sister, and there were no concerns regarding finances, housing, domestic violence, or substance misuse.

### 2.1. Physical Health, Mental Health, and Functional/Dissociative Seizures

Childhood physical health diagnoses included asthma, gastrointestinal issues, and Tourette’s syndrome, which was diagnosed as a young child. The patient reported that his mother has consistently expressed anxiety regarding his health, leading to frequent consultations with his general practitioner and other healthcare professionals. He explained that whilst such interactions offered a problem-focused experience of receiving care, he also received dedicated attention and support, due to adopting the role of a ‘sufferer’ or ‘victim’. The patient reported that he believed that he received several unnecessary medical treatments, tests, and physical health diagnoses as a child. Aged 12 years, the patient was referred to child and adolescent mental health services due to concerns regarding low mood and anxiety. He completed an ineffective course of individual cognitive behavioural therapy (CBT). He was re-referred at 13 years of age due to anxiety, low mood, and self-harm. The family were offered and declined family therapy. The patient was prescribed fluoxetine and then sertraline, both with limited effects, before stopping all psychotropic medication. At the time of this study, the patient was not prescribed any psychiatric medication. At approximately 14 years of age, the patient began experiencing seizures, and, following assessment by a consultant neurologist, he was diagnosed with non-epileptic attack disorder. Due to the severity of functional/dissociative seizures, the patient had stopped attending at college 1 month before starting CAT, and therefore he was not in education, employment, or training. At screening, the patient’s primary concerns were dissociative seizures and low self-esteem. He described poor appetite, sleep, low self-worth, and hopelessness. At the start of the psychological intervention, the dissociative seizures were occurring at least once a week and usually preceded by relational conflict (e.g., an argument with peer/girlfriend/family). Seizures varied in terms of frequency, duration and intensity. The symptoms of the seizures were as follows: each seizure lasted up to 15 min including motor weakness, slumped posture, and being unresponsive. Functional dissociative seizures could occur irrespective of context.

### 2.2. Design

This study follows best guidance on reporting a SCED [SCRIBE] [25]. The participant provided written consent for this study to be conducted and reported. The SCED was a mixed-methods A-B-C design with a 5-week follow-up and a patient interview 13 weeks after completing therapy. The idiographic measures were the target problems (TPs) and associated target problem procedures (TPPs) and change in these measures was theoretically mapped onto the three phases of CAT (i.e., creating an A-B-C design). The three CAT phases were reformulation (2 sessions), recognition (3 sessions), and revision (3 sessions), with a single follow-up (FU) session conducted at 5 weeks. TPs and TPPs were rated at each session to track whether self-awareness had increased (i.e., recognition) and change had also been enabled (i.e., revision). Nomothetic outcome measures were administered at session 1, session 8, and at follow-up. An interview was conducted with the patient 13 weeks after therapy was completed. See Table 1 for a summary of the timing of the measures and the design of this study.

### 2.3. Idiographic Outcome Measures and Analysis Strategy

Two TPs and associated TPPs (one trap and one dilemma) were rated at each session for recognition (0—‘cannot see the pattern’ to 100—‘spotting it really well’) and revision (0—‘have not been able to change it’ to 100—‘changing it very effectively’. The first TP was ‘my low self-esteem trap’ with a TPP of *“I feel worthless and can’t get what I want because things turn bad or go wrong for me. I therefore feel on edge and that all is hopeless. Because I think this, every time I try to do things it feels more draining, and I feel that there is ‘no point in trying’ and I give in. This confirms my feels that everything is hopeless, and I am worthless, and I think of suicide and self-harm”*. The second TP was ‘my bottling up feelings versus explosive reactions dilemma’ with a TPP of *“Because of past experiences of being angrily rejected, I have had few good experiences of being listened to and cared for in a way that feels helpful, and I am fearful of being rejected. I therefore assume this will happen in most of my relationships and I therefore keep my thoughts and feelings to myself to in an avoid conflict and possible rejection. This however means that my feelings build up. As my feelings don’t go away, they become overwhelming and can result in me having a seizure or exploding in a way that I let all my frustrations and feelings out in one big go. This all leaves me feeling alone, rejected, and worthless”.* The effectiveness of CAT on the two TPs and associated TPPs were assessed using three nonoverlap statistics: the percentage of data points exceeding the median [PEM [26]], the percentage of all nonoverlapping data [PAND [27]], and nonoverlap of all pairs [NAP [28]]. The nonoverlap outcomes were interpreted in line with available guidance [29] so that <70% on PEM, PAND, and NAP indicates questionable/ineffective treatment, 70–90% indicated a moderately effective treatment, and >90% indicated that a highly effective treatment had taken place.

### 2.4. Nomothetic Outcome Measures and Analysis Strategy

Two self-report nomothetic outcome measures (DES-II and RCADS) were completed before session 1, at the end of session 8, and at the follow-up session. Two other self-report nomothetic outcomes measures (SRS and YP-CORE) were completed at each session. Change in the DES-II, YP-CORE, and the RCADS was assessed using reliable and clinically significant change criteria [RCSC [30]]. RCI assesses whether the degree of change on a nomothetic measure is beyond random measurement error or chance; RCI values ≥ 1.96 are a significant and reliable change. Nomothetic measures were also assessed for clinical change (i.e., when scores shifted from a clinical adolescent to a non-clinical adolescent population). When the intake score is in the clinical range, there are four categories of possible outcomes when using the RCI and clinical change simultaneously: recovered, improved, unchanged, and deteriorated.

The Dissociative Experiences Scale-II (DES-II) assesses the severity of dissociative experiences and consists of 28 Likert-type scales ranging from 0% (never) to 100% (always). The total DES-II score is the mean of all items. The DES-II has been shown to have high reliability (Cronbach’s α = 0.95), and strong evidence of convergent, discriminant, and criterion validity [31]. Normative data [32] is available across various clinical and non-clinical samples, with means and standard deviations as follows: student/adolescent: 14.27 (SD = 11.54); general psychiatric patient: 16.66 (SD = 16.41); history of abuse: 29.17 (SD = 20.99); personality disorders: 19.61 (SD = 16.24); PTSD: 32.01 (SD = 19.18); and dissociative disorders: 41.22 (SD = 21.99). The cut-off score for identifying dissociation is 30, and the reliable change score is 21.99.

The Revised Children’s Anxiety and Depression Scale (RCADS) has 47 items and assesses anxiety and depression symptoms in 8- to 18-year-old children and adolescents. The RCADS yields subscale scores for various anxiety disorders (separation anxiety, social phobia, obsessive-compulsive disorder, panic disorder, generalized anxiety disorder) and depression. The RCADS has been found to have good internal consistency, with the Cronbach’s alpha for each subscale being as follows: separation anxiety α = 0.78; social phobia: α = 0.87; obsessive-compulsive disorder: α = 0.82; panic disorder: α = 0.88; generalised anxiety disorder: α = 0.84; major depressive disorder = α = 0.87 [33]. A t-score of 70 is the caseness cut-off score [33], and the reliable change index criteria score is 14.91 for generalised anxiety, 18.29 for panic disorder, 22.95 for separation anxiety, 13.99 for social phobia, and 17.73 for depression.

Young Person’s CORE (YP-CORE) is a 10-item measure that assesses psychological distress in those 11–18 years old and has subscales of well-being, problems/symptoms, and life/social functioning. The YP-CORE has good internal reliability, with a Cronbach’s alpha of 0.8 [34]. The RCI is a score change >8.33, and the clinical cut-off is a score of 14+.

The Session Rating Scale is a four-item measure assessment of the quality of the therapeutic alliance in that session. The SRS measures (a) respect and understanding; (b) relevance of goals and topics; (c) client–practitioner fit; and (d) overall alliance [35]. The SRS has high internal consistency, and test–retest reliability ranges from 0.54 to 0.70 [36].

### 2.5. Change Interview

Thirteen weeks after completing CAT, the patient was interviewed on the telephone with the Change Interview by an Assistant Psychologist who was uninvolved with the therapy to reduce bias. The Change Interview is a semi-structured interview that can be conducted mid-treatment, at the end of treatment, or at follow-up [37]. The Change Interview focuses on whether change has occurred during therapy, and, if change has occurred, then what the changes are and whether they are connected to the therapy (or not) delivered. The Change Interview therefore does not assume that therapy has been helpful and adopts a sceptical stance [38]. Each change was rated (1–5) for how expected the change was, the importance of the change, ability to apply change in everyday life, and likelihood of this change without the therapy. The patient was also asked to provide 3 words to describe the psychological work completed and rate on a scale of 0–100 how supportive and effective the psychological input was.

### 2.6. Treatment

Cognitive analytic therapy (CAT) is a brief, structured, transdiagnostic and integrative psychotherapy commonly delivered in the NHS typically in the treatment of complex and enduring mental health problems [18]. CAT is a time-limited psychotherapy offered in either 8, 16, or 24 weekly sessions, with treatment duration matched to patient complexity and preference [18]. Meta-analytic evidence highlights both the effectiveness and acceptability of CAT across a range of diagnoses and clinical contexts [39,40]. In the context of FDS, there is evidence of CAT being effective with dissociative disorders [41,42]. The evidence base for CAT in the treatment FND comprises two uncontrolled case reports of outpatient therapy with adults [19,20] and an N = 6 case series [43] to show that 4 patients completed it, and there were reductions to psychological distress. The rationale for offering CAT as opposed to retraining and control therapy (ReACT) or CBT was due to (a) CBT being previously offered and being ineffective, and (b) there were strong relational elements to the case presentation (e.g., the attention received when adopting ’sufferer’ or ‘victim’ role and the trigger often being relational conflict)—and CAT is a relational psychotherapy [18].

CAT has a three-phase theoretical structure of reformulation, recognition, and revision. These phases aim to identify, monitor, and then change the idiographic target problems (TPs) and target problem procedures (TPPs) of the case [18]. TPPs are either snags (self- sabotage), traps (vicious circles), or dilemmas (rigid either/or choices). In the context of FDS, this three-phase structure would aim to develop and share an understanding of the developmental origins and current maintainers of the FDS (reformulation), enabling the patient to then better recognise the roles and patterns maintaining the FDS (recognition), and finally help the patient to make active therapeutic change to the FDS (revision).

Formulating the role of childhood traumas and attachment difficulties is important because there is meta-analytic evidence that previous maltreatment and stressful life events are substantially more common in FND compared to healthy and patient controls [44]. CAT models how these factors then continue to influence relationships with the self and others in the present day [18]. The competency model for CAT [45] defines the ways therapists should move through the three phases, and this emphasises ongoing management of ‘enactments’ in the therapeutic relationship (i.e., when the patient is relating to the therapist in ways that mirror previously important developmental relationships [18]).

Treatment was delivered in the United Kingdom in a child and adolescent mental health service provided by the National Health Service. The therapist was a male clinical psychologist and CAT therapist under monthly clinical supervision from a CAT practitioner. Following a referral and a screening session, the client was allocated to the ‘Sheffield model’ version of eight-session CAT [46]. All sessions were weekly, 50 min in length, and conducted by the same therapist irrespective of the study phase. Three of the eight sessions were rearranged (due to sessions being missed due to dissociation), but the patient did attend all eight sessions. The treatment protocol was a session-by-session guide designed for the RELATE feasibility randomised control trial of CAT for self-harm [47].

The first two sessions were focal to assessment (e.g., taking a history), they did not contain any treatment elements, and the narrative reformulation (NR) was shared at session three. The NR identified the developmental origins of the FDS, stated the TPs and TPPs, and highlighted possible enactments in the therapeutic relationship. The main components of CAT therapy (i.e., NR, sequential diagrammatic reformulation [SDR] and goodbye letters exchanged between client and therapist at termination) were present [18], and each component was reviewed within clinical supervision. The revision stage of CAT concentrated on change, and changes were identified and then labelled as ‘exits’ on the SDR. The patient was encouraged to practice the exits as homework tasks. The exits were concerned with creating a better model of self-care and better management of emotions and were specifically finding and creating better support from people, engaging with hobbies, listening to music, better conflict management, having a plan, committing to actions with purpose, and noticing (but not judging) the urge to be ill. Overall, the aim was practicing being seen, heard, and noticed in the world in a healthy manner.

### 2.7. Results and Outcomes

The results have four sections (a) plotting and nonoverlap analysis of the two TPs and associated TTPs, (b) analysis of reliable and clinically significant change in the nomothetic outcomes measures, (c) plotting and analysis of the impact of each session with the SRS, and (d) identifying changes associated with the therapy and the personal significance of these via the Change Interview.

### 2.8. Changes in Target Problems and Target Problem Procedures

**Figure 1:**
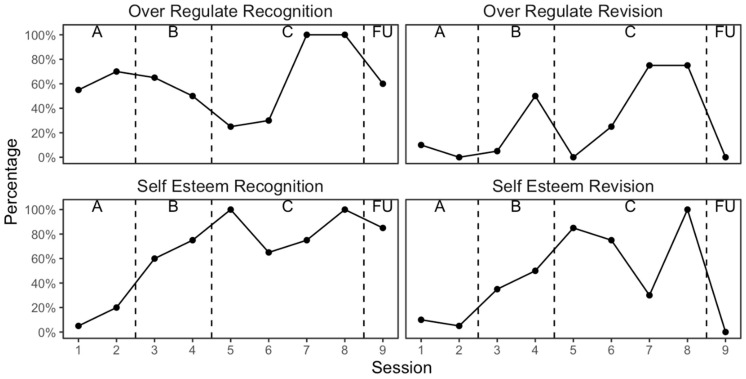
Change in target problems (TPs) and target problem procedures (TPPs).

Figure 1 reports the plots for the recognition and revision sessional ratings of the two TPs and TPPs, and Table 2 reports the nonoverlap effectiveness analysis. There was better awareness and management of the emotion dilemma during therapy, but this was not retained at follow-up. The nonoverlap results would index that an effective intervention had taken place, but when the therapy was compared to follow-up, this showed evidence of relapse in terms of the patient’s ability to effectively keep revision going during the follow-up period. There was also better awareness and management of the self-esteem trap during therapy, and again this was not retained at follow-up in terms of revision abilities. The nonoverlap results would index that an effective intervention had taken place, but when the therapy was compared to follow-up, this showed evidence of relapse in relation to revision.

### 2.9. Nomothetic Outcomes

**Figure 2:**
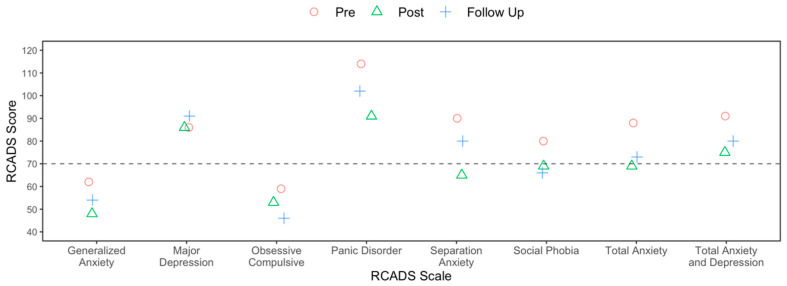
Revised Children’s Anxiety and Depression Scale (RCADS) and pre, post and follow-up.

**Figure 3:**
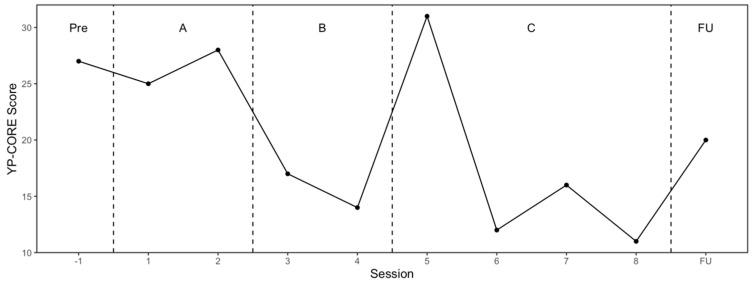
Young Person’s CORE: session scores.

The RCADS (pre–post and follow-up) and the sessional YP-CORE results are presented in Figure 2 and Figure 3, respectively. On the RCADS there was a reliable pre–post reduction in generalised anxiety and panic, and the social phobia follow-up score was below caseness (t-score < 70). There was no evidence of relapse on the RCADS. On the YP-CORE there was reliable and clinically significant reduction in psychological distress on the pre-post comparison, but then a reliable deterioration during the follow-up period. The DES-II was above the clinical cut-off (i.e., 30) at session 1 (score = 68), session 8 (score = 42), and the follow-up session (score = 38), but there was a reliable pre–post reduction in dissociation that was maintained at follow-up.

### 2.10. Session Impact

**Figure 4:**
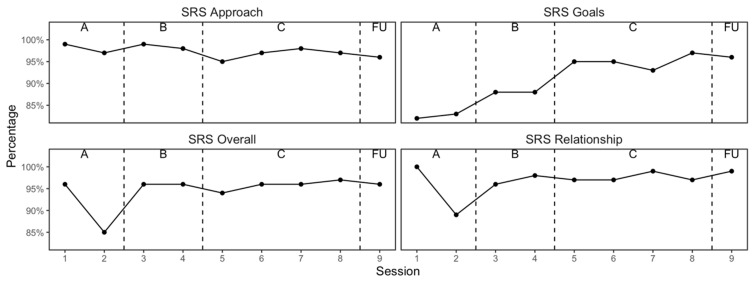
Session Rating Scale (SRS) scores at each session.

Figure 4 plots the impact of each session in terms (a) respect and understanding, (b) relevance of the goals and topics of the session, (c) patient–therapist fit, and (d) the overall alliance. These outcome graphs show that the alliance was initially high, and this was maintained throughout the therapy. The sessions became increasing goal-focused and the relevance of the session content increased as the patient progressed through the phases of CAT.

### 2.11. Change Interview Results 

The patient described the CAT as “helpful, open and insightful” and rated the intervention as supportive (95/100), effective during treatment (86/100), and effective at follow-up (60/100). The four idiographic changes are detailed in Table 3, and changes were rated as surprising, personally important, practical, and unlikely without the therapy. The seizures had stopped during the follow-up period, and, at the time of the Change Interview, the patient had been seizure-free for 8 weeks.

## 3. Discussion

The treatment of adolescent FND is a clinical challenge [16], and there have been no previous descriptions or evaluations of integrative psychotherapy. More specifically, there have been no previous controlled evaluations of the effectiveness and durability of CAT for FND in adolescents, and no previous SCEDs of any other psychotherapies with this clinical population. This current study therefore evaluated the effectiveness of brief eight-session CAT in a case of an adolescent with FDS and evaluated the outcomes using a mixed-methods SCED. The patient was vulnerable to FDS due to the role of previous trauma impacting on self–self, self–other, and other–self relationships [18]. The previous CBT would not have enabled the patient to process the previous trauma, as it would have worked exclusively in the here and now. CAT would appear to offer a reasonable treatment offer for adolescents with FND where there is previous trauma affecting current functioning.

Combining both qualitative and quantitative approaches enabled a complex tapestry of outcomes across multiple spheres to be created and considered. The evaluation of the overall picture was one of a partially effective therapy. The SCED design employed (i.e., A-B-C-FU) offered greater internal validity than the traditional bi-phasic (A-B) and quasi-experimental designs commonly used in routine practice [48]. This study also benefitted from patient interviewing, therefore making it a mixed-methods SCED, which is also innovative as an evaluation approach.

The verdict of CAT being a partially effective intervention for the adolescent with FDS was due to there being both positive and negative outcomes. Positively, this was based on the evidence of the Change Interview (seizures having stopped for 2 months, and this change being attributable to the therapy), effectiveness in the increased recognition and revision of the TP/TPPs during therapy, sessions becoming increasingly goal-focused, a reliable pre–post improvement in dissociation (DES-II), and reliable pre–post and clinically significant improvements to psychological wellbeing (YP-CORE). The cessation in seizures by the patient was seen as a product of CAT enabling better emotion regulation, better self-care, and better relationships. Seizure cessation also enabled more effective behavioural engagement with life. CAT is a focal form of psychotherapy, and the effective recognition and revision of the TP/TPPs is the presumed mechanism of action for the outcomes on the nomothetic measures. Careful preparation for the termination of therapy did not prevent some outcomes from deteriorating over the follow-up period. Negatively, this was based on the evidence of relapse at follow-up in terms of the revision of the TP/TPPs, a reliable deterioration in psychological well-being (YP-CORE), and the fact that none of the nomothetic outcome measures were in the community range at follow-up. The patient was prepared for the follow-up via the goodbye letter that is a routine part of CAT, which also supports relapse prevention [18]. Therefore, efforts were made to prepare the patient for the follow-up period. All sessions were attended, indicating an acceptable approach, and this is important as there is meta-analytic evidence that 50% of adolescents drop out of treatment in routine services [49]. CAT has been shown to have differentially high acceptability rates [40].

In terms of limitations, outcome data was all self-reported, and this limits confidence due to the potential for social desirability bias. This could have been corrected through the additional use, for example, of the parent version of the RCADs at pre–post and follow-up [50]. The robustness of the design would have been improved through the introduction of a withdrawal phase, although the ethics of removing a therapy that is effective needs to be carefully considered (see [51] for an example of a withdrawal design using CAT). The methodology would also have been improved through daily monitoring of seizure frequency, as has been achieved in a recent CAT SCED in adult FND [21]. Adding in a generalisation nomothetic measure [52] would have been useful. The use of an adolescent-specific dissociation scale such as the Adolescent Dissociative Experiences Scale [53] may have been more sensitive to the context. The patient was selected for brief eight-session CAT, although it is acknowledged that the 16- or 24-session version of the approach may have better insulated the patient from the relapse that occurred in the follow-up. The follow-up period was relatively brief at 5 weeks, so future studies should explore longer follow-up periods. The clinical competency of the intervention delivered could have been assessed via the Competency in CAT measure [54]. Future SCEDs with adolescent patients with FND may also seek to use cross-over designs where there could be random allocation to CAT versus CBT [55], which would enable an evaluation of outcomes produced by each approach to be compared to each other and the baseline.

## 4. Conclusions

In this innovative and methodologically unique study, outpatient CAT was found to be a partially effective treatment for an adolescent with FDS from an analysis of the qualitative and quantitative outcome matrix. Treatment acceptability was good, with all sessions being attended. This case report encourages more outcome-based research to be conducted with CAT for adolescent FND. SCED would be a useful methodology in building more evidence, before progressing onto feasibility and efficacy randomised controlled trials.

## Figures and Tables

**Table 1:** The design of this study and the timing of the measures.

Session 1 (Reformulation A SCED Phase)	Session 2 (Reformulation A SCED Phase)	Session 3 (Recognition B SCED Phase)	Session 4 (Recognition B SCED Phase)	Session 5 (Recognition B SCED Phase)	Session 6 (Revision C SCED Phase)	Session 7 (Revision C SCED Phase)	Session 8 (Revision C SCED Phase)	Follow-Up (Revision)
SRS	SRS	SRS	SRS	SRS	SRS	SRS	SRS	SRS
YP-CORE	YP-CORE	YP-CORE	YP-CORE	YP-CORE	YP-CORE	YP-CORE	YP-CORE	YP-CORE
DES-II							DES-II	DES-II
R-CADS							R-CADS	R-CADS
TP/TPP recognition rating	TP/TPP recognition rating	TP/TPP recognition rating	TP/TPP recognition rating	TP/TPP recognition rating	TP/TPP recognition rating	TP/TPP recognition rating	TP/TPP recognition rating	TP/TPP recognition rating
TP/TPP recognition rating	TP/TPP recognition rating	TP/TPP recognition rating	TP/TPP recognition rating	TP/TPP recognition rating	TP/TPP recognition rating	TP/TPP recognition rating	TP/TPP recognition rating	TP/TPP recognition rating
								qualitative: change interview conducted 13 weeks after CAT completion

**Table 2:** Nonoverlap analysis of the effectiveness of the change in target problems (TPs) and target problem procedures (TPPs).

	NAP (95% CI)	PAND	PND	PEM
Reformulation (A) vs. Recognition (B)				
Self-Esteem—Recognition	100% (100% to 100%)	100%	100%	100%
Self-Esteem—Revision	100% (100% to 100%)	100%	100%	100%
Over-Regulate—Recognition	17% (2% to 69%)	60% 60	0%	33%
Over-Regulate—Revision	58% (17% to 90%)	60%	33%	50%
Reformulation (A) vs. Revision (C)				
Self-Esteem—Recognition	100% (100% to 100%)	100%	100%	100%
Self-Esteem—Revision	100% (100% to 100%)	100%	100%	100%
Over-Regulate—Recognition	67% (21% to 93%)	80%	67%	67%
Over-Regulate—Revision	100% (100% to 100%)	100%	100%	100%
Reformulation (A) vs. Follow-Up (FU)				
Self-Esteem—Recognition	100% (100% to 100%)	100%	100%	100%
Self-Esteem—Revision	0% (0% to 0%)	67%	0%	0%
Over-Regulate—Recognition	50% (8% to 92%)	67%	0%	0%
Over-Regulate—Revision	25% (2% to 84%)	67%	0%	0%
Recognition (B) vs. Follow-Up (FU)				
Self-Esteem—Recognition	33% (4% to 85%)	75%	0%	0%
Self-Esteem—Revision	100% (100% to 100%)	100%	100%	100%
Over-Regulate—Recognition	33% (4% to 85%)	75%	0%	0%
Over-Regulate—Revision	83% (23% to 99%)	75%	0%	100%

Abbreviations: NAP nonoverlap of pairs; PAND percentage of all nonoverlapping data; PND percentage of overlapping data; PEM percentage of data points exceeding the median.

**Table 3:** Change interview results.

Description of Personal Changes	Change Was: 1—Expected by Me, 2—Somewhat Expected by Me, 3—Neither Expected or Surprising, 4—I Was Somewhat Surprised, 5—I Was Very Surprised by.	Importance of This Change to Me: 1—Not at All, 2—Slightly, 3—Moderately, 4—Very, 5—Extremely.	Ability to Apply This Change in Everyday Life: 1—Can’t Apply, 2—Can Somewhat Apply, 3—Can Apply Some Aspects, 4—Can Apply Most Aspects, 5—Can Apply All Aspects.	Likelihood of This Change Without Therapy 1—Very Unlikely, 2—Somewhat Unlikely, 3—Neither, 4—Somewhat Likely, 5—Likely.
No seizures for two months	4	5	5	1
Stopped ‘exploding’ emotionally and feel calmer	1	4	3	2
Taking more time to be with self and not constantly needing to be around people	4	5	4	3
Better model of relationships	2	4	4	2

## Data Availability

The original contributions presented in this study are included in this article; further inquiries can be directed to the corresponding author.

## Abbreviations

The following abbreviations are used in this manuscript:

CAT	Cognitive analytic therapy
SCED	Single case experimental design
SES	Session Evaluation Scale
FDS	Functional dissociative seizure
FND	Functional neurological disorder
YP-CORE	Young Persons Clinical Outcome Routine Evaluation
RCADS	Revised Children’s Anxiety and Depression Scale
DES-II	Dissociative Experiences Scale-II
NR	Narrative reformulation
SDR	Sequential diagrammatic reformulation
TP	Target problem
TPP	Target problem procedure
